# Recent trends in hospitalizations for myelodysplastic syndromes and polycythemia vera in England and Wales

**DOI:** 10.1097/MD.0000000000049861

**Published:** 2026-07-24

**Authors:** Alaa A. Alsharif, Abdallah Y. Naser, Richard Ofori-Asenso

**Affiliations:** a Department of Pharmacy Practice, College of Pharmacy, Princess Nourah bint Abdulrahman University, Riyadh, Saudi Arabia; b Department of Applied Pharmaceutical Sciences and Clinical Pharmacy, Faculty of Pharmacy, Isra University, Amman, Jordan; c Cardiovascular, Renal, Metabolism Evidence, BioPharmaceuticals Medical, AstraZeneca, Cambridge, UK.

**Keywords:** admissions, England, myelodysplastic, polycythemia vera, Wales

## Abstract

Myelodysplastic syndromes (MDS) are among the most common hematological malignancies. Polycythemia vera (PV) is a myeloproliferative disorder that causes the neoplastic proliferation of hematopoietic progenitor cells. Limited data exist on the population-level burden of MDS and PV globally. This ecological study utilized publicly available data from the Hospital Episode Statistics database in England and the Patient Episode Database for Wales for the period from April 1999 to April 2020. The hospital admission rate was 71.6% (from 72.62 [95% confidence interval [CI]: 71.89–73.35] in 1999 to 124.60 [95% CI 123.71–125.50] in 2020 per 100,000 persons, trend test, *P* < .05). MDS and PV-related admissions increased by 85.3% and 24.2%, respectively. In males, the admission rate increased by 86.1% (from 88.36 [95% CI: 87.21–89.52] in 1999 to 164.48 [95% CI: 163.02–165.94] in 2020 per 100,000 persons), whereas in females, there was an increase of 48.5% (from 57.62 [95% CI: 56.71–58.53] in 1999 to 85.55 [95% CI: 84.51–86.60] in 2020 per 100,000 persons). During the past 21 years, there has been a substantial rise in MDS and PV-related hospital admissions in England and Wales. Further studies are needed to establish the population factors contributing to these trends in order to inform public health measures.

## 1. Introduction

Blood cancers that originate from bone marrow, for example, myelodysplastic syndromes (MDS) and myeloproliferative neoplasms (MPNs), are a complicated and constantly changing problem for modern medical care. Among these cancers, polycythemia vera (PV) and MDS are 2 extreme points of a shared biological spectrum: 1 marked by overproduction of blood cells and the other by hematopoiesis inefficiency. The 2 disorders are associated with a high risk of thrombotic events, progression, and transformation to acute leukemia, causing a substantial burden on patients and global healthcare systems^[1,2]^.

Prior population-based studies indicate that these conditions, which are still relatively rare, are increasingly being recognized, especially among elderly individuals. According to data obtained from national registries in the United States, the annual age-adjusted incidence rates were 3.3 per 100,000 for MDS and 2.1 per 100,000 for chronic MPNs, with a notable increase in rates with age.^[2]^ A mutation in the JAK2 gene is the primary cause of PV. This mutation leads to overproduction of red blood cells and an increased risk of thrombosis and myelofibrosis.^[3]^ Regardless of advancements in diagnosis and treatment, PV still presents clinical and epidemiological concerns.

The literature has been investigating the management of PV across various healthcare systems. A comprehensive study in Taiwan reported a prevalence of 10.8 cases per 100,000 and observed significant variation in the use of standard therapies, such as hydroxyurea and phlebotomy.^[4]^ About 1100 new cases are diagnosed in the United Kingdom (UK) yearly; however, compliance with national guidelines differs from place to place, and achieving the best hematocrit control is still a challenge for many patients.^[3,5]^ A prior study in the UK indicates significant gaps in disease management and follow-up, with fewer than 60% of patients maintaining their hematocrit levels below the recommended limit.^[3]^

Likewise, MDS is still a major contributor to hospital use and morbidity, mainly in an elderly population. The chronic and frequently progressive pattern of MDS causes repeated hospital admissions (HAs) for transfusion support and management for infection and disease complications. Although incidence and survival have been extensively characterized, trends in HA, which reflect both the clinical burden and the use of healthcare resources, have not been discussed, particularly in the National Health Service context. Knowledge of changes in HAs for MDS and PV over time is valuable in identifying shifts in disease recognition, management practices, and outcomes. Besides, it can aid in informing service planning and decision-making for resource allocation in hematology care. Hence, this research aims to evaluate changes in HA rates for MDS and PV in England and Wales (EW).

## 2. Methods

### 2.1. Study sources and the population

This was an ecological study using publicly available data extracted from the Hospital Episode Statistics (HES) database in England^[6]^ and the Patient Episode Database for Wales (PEDW) for the period between April 1999 and April 2020.^[7]^ Multiple studies used these 2 databases to study the epidemiology of hospitalization for different diseases and age groups.^[8–10]^ The HES and PEDW databases contain HA data for patients with PV and MDS from all age groups, which are subdivided into 4 categories: below 15 years, 15 to 59 years, 60 to 74 years, and 75 years and above. We identified PV and MDS-related HAs using the Tenth Revision of the International Statistical Classification of Diseases and Related Health Problems (ICD-10) 5th Edition (used by the National Health Service to classify diseases and other health conditions) recorded as the primary diagnosis, where the main diagnosis and cause of admission were recorded.^[11]^ All diagnostic codes for PV and MDS (D45–D46) were used to identify all HAs related to different types of PV and MDS in EW. HES and PEDW data are checked regularly to ensure their validity and accuracy.^[6,12]^ To calculate the yearly HA rate for PV and MDS, we collected mid-year population data for the period between 1999 and 2020 from the Office for National Statistics.^[13]^ Each patient record is defined as a continuous period of care (episode) that is administered at a single hospital provider within a specific consultant specialty. Consequently, a new episode is reported when the same patient is transferred to a different provider or to another consultant.^[14]^ Therefore, a single HA could comprise multiple finished consultant episodes.

PV and MDS are recorded in the above-mentioned 2 medical databases using disease-specific ICD-10 codes (D45 and D46, respectively). This facilitates consistent case ascertainment for the included data and enables accurate examination of the hospitalization trends. Other chronic MPNs, including essential thrombocythemia and primary myelofibrosis, are coded under the nonspecific category D47 “Other neoplasms of uncertain behavior of lymphoid, hematopoietic and related tissue,” which also includes other conditions of uncertain behavior, which would reduce the specificity of the coding and the validity of trend estimates.

### 2.2. Statistical analysis

Rates of HAs with 95% confidence intervals (CIs) were calculated using the finished consultant episodes of PV and MDS-related admissions divided by the mid-year population. We used the chi-squared test to assess the difference between the HA rates between 1999 and 2020. The trend in HAs was assessed using a Poisson model. All analyses were conducted using SPSS version 25 (IBM Corp., Armonk, NY).

## 3. Results

**Figure 1.**
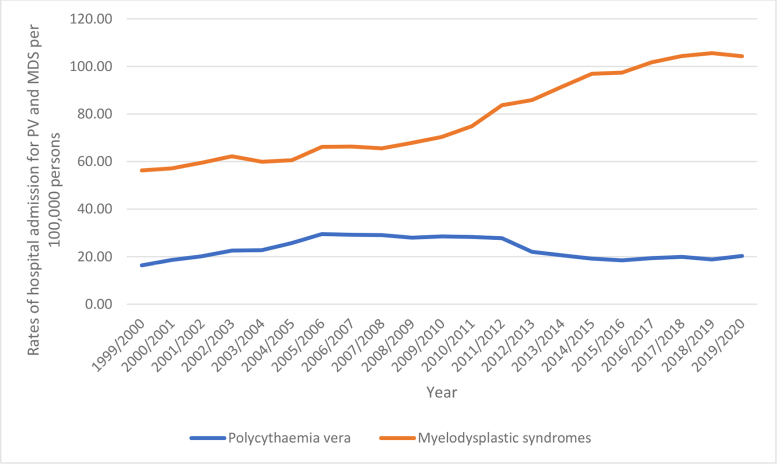
Rates of HA for PV and MDS in EW from 1999 to 2020. EW = England and Wales, HAs = hospital admissions, MDS = myelodysplastic syndromes, PV = polycythemia vera.

The total annual number of HAs for PV and MDS increased by 96.5% from 37,865 in 1999 to 74,413 in 2020, representing an increase in the HA rate of 71.6% (*P* < .05). Besides, MDS accounted for 77.4% of total HAs. Over the study duration, the HA rate for MDS and PV increased by 85.3% and 24.2%, respectively (Table 1, Fig. 1).

**Table 1:** Total HAs for PV and MDS from 1999 to 2020 in EW.

Categories	Total	PV	MDS
*N*	1,190,522	269,428	921,094
Percentage from total		22.6%	77.4%
HAs rate in 1999 per 100,000 persons (95% CI)	72.62 (71.89–73.35)	16.35 (16.00–16.70)	56.27 (55.63–56.92)
HAs rate in 2020 per 100,000 persons (95% CI)	124.60 (123.71–125.50)	20.31 (19.95–20.67)	104.30 (103.48–105.12)
Percentage change from 1999 to 2020	71.6%	24.2%	85.3%

CI = confidence intervals, HAs = hospital admissions, MDS = myelodysplastic syndromes, *N* = number of patients, PV = polycythemia vera.

### 3.1. Admissions rate by age group

**Figure 2.**
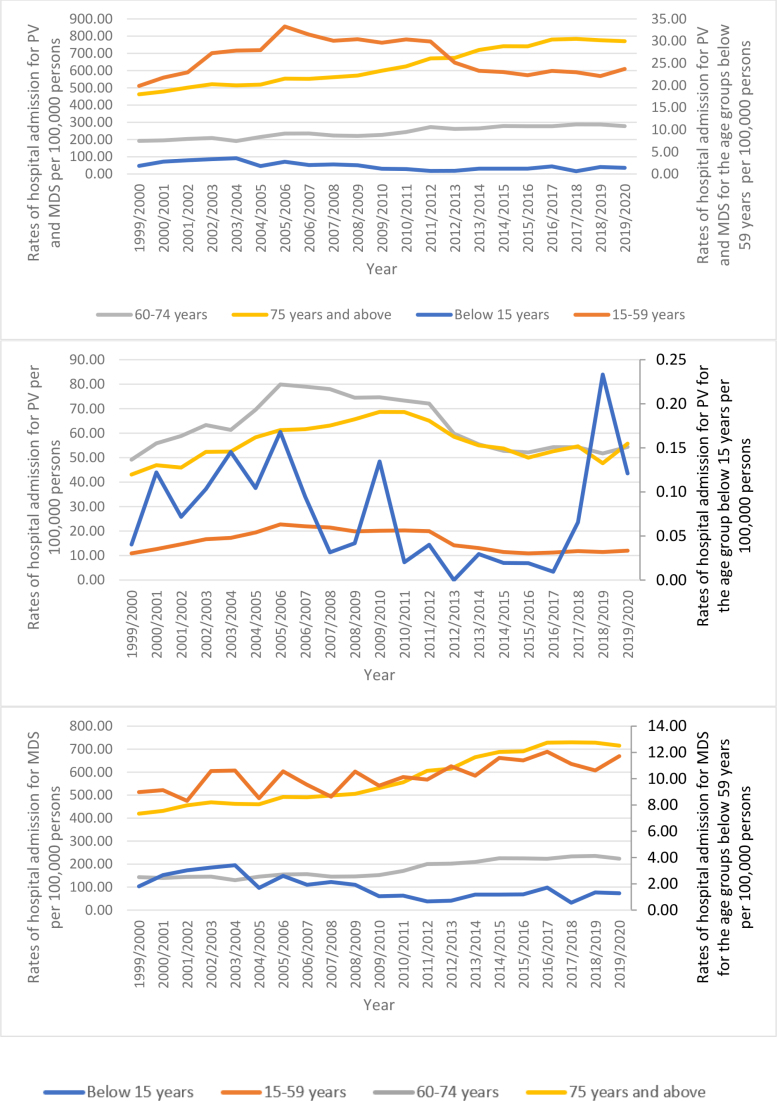
Rates of HA for PV and MDS in EW stratified by age group. EW = England and Wales, HAs = hospital admissions, MDS = myelodysplastic syndromes, PV = polycythemia vera.

Regarding age group diversity for PV and MDS HA, HA was directly related to age; the age group 75 years and above accounted for the most HA episodes (49.6%). Rates of HA for PV and MDS among patients aged below 15 years decreased by 23.9%; however, among patients in all other age groups, it increased (Table 2, Fig. 2). Furthermore, the rate of HA for MDS was directly related to age (more common among the age group 75 years and above). However, the rate of HA for PV was more common among the age groups: 60 to 74 years, 75 years and above, 15 to 59 years, and below 15 years, respectively (Fig. 2).

**Table 2:** Total HAs for PV and MDS from 1999 to 2020 in EW, stratified by age group.

Categories	N (percentage from total)	HAs rate in 1999 per 100,000 persons (95% CI)	HAs rate in 2020 per 100,000 persons (95% CI)	Percentage change from 1999 to 2020
Below 15 years	3761 (0.3%)	1.85 (1.58–2.11)	1.41 (1.18–1.63)	–23.9%
15–59 years	182,002 (15.3%)	19.88 (19.39–20.38)	23.72 (23.21–24.24)	19.3%
60–74 years	413,878 (34.8%)	192.23 (188.97–195.49)	277.91 (274.54–281.28)	44.6%
75 years and above	588,958 (49.6%)	462.55 (455.83–469.27)	770.91 (763.37–778.45)	66.7%

CI = confidence intervals, HAs = hospital admissions, MDS = myelodysplastic syndromes, *N* = number of patients, PV = polycythemia vera.

### 3.2. Admissions rate by gender

**Figure 3.**
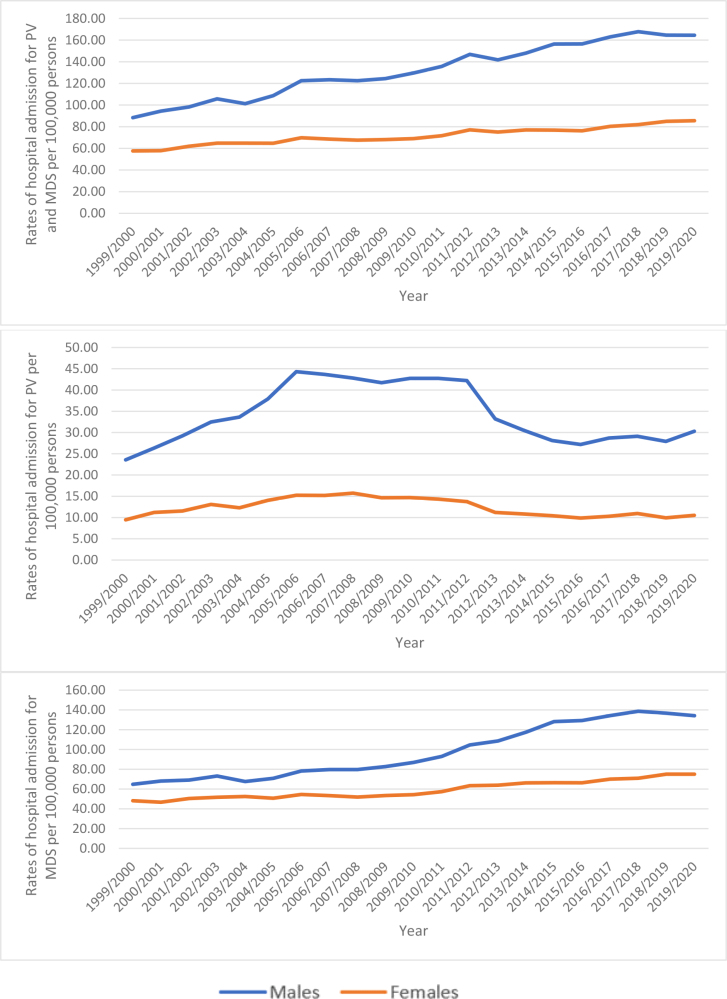
Rates of HA for PV and MDS in EW stratified by gender. EW = England and Wales, HAs = hospital admissions, MDS = myelodysplastic syndromes, PV = polycythemia vera.

Regarding gender diversity, males accounted for 64.2% of the total number of HA episodes for PV and MDS, or 763,833 HA episodes, with a mean of 36,373 episodes per year. The rate of HA for PV and MDS among males increased by 86.1%; however, among females, it increased by 35.8% (Table 3, Fig. 3). Moreover,rates of HA for PV and MDS were higher among males compared to females (*P* < .05) (Fig. 3).

**Table 3:** Total HAs for PV and MDS from 1999 to 2020 in EW, stratified by gender.

Cate gories	N (percentage from total)	HAs rate in 1999 per 100,000 persons (95% CI)	HAs rate in 2020 per 100,000 persons (95% CI)	Percentage change from 1999 to 2020
Males	763,833 (64.2%)	88.36 (87.21–89.52)	164.48 (163.02–165.94)	86.1%
Females	426,644 (35.8%)	57.62 (56.71–58.53)	85.55 (84.51–86.60)	48.5%

CI = confidence intervals, HAs = hospital admissions, MDS = myelodysplastic syndromes, N = number of patients, PV = polycythemia vera.

## 4. Discussion

Our study found that from 1999 to 2020, in EW, the total annual number of HAs for PV and MDS increased by 96.5%, representing a 71.6% increase in the HA rate, from 72.62 to 124.60 per 100,000 population (*P* < .05). Besides, the rates of HAs for MDS and PV increased by 85.3% and 24.2%, respectively. Consistent with these results, a previous study conducted in EW from 1999 to 2019 reported that the total annual number and rate of HAs related to neoplasms, including MDS and PV, increased by 66.7% and 46.2%, respectively.^[15]^ The rise in HAs could be attributed to advances in diagnosis and treatment, the rise in incidences of MDS and PV, population growth, and aging.

The UK population is both growing^[16]^ and aging,^[17]^ with about 19% of the population aged 65 and above.^[17]^ Besides, the estimated annual incidence of newly diagnosed patients with MDS and PV in the UK is 2160 and 1140, respectively.^[18]^ In terms of PV, patients with PV suffer from many complications related to their disease, including thrombotic events, disability, and prolonged hospital stays, which lead to increasing healthcare costs.^[19]^ Regardless, several prior studies emphasize that early diagnosis, interventions, and/or efficacious management of PV can prevent PV-related complications and risk, as well as improve patient survival, life expectancy, and/or quality of life.^[20–25]^ Thus, early diagnosis, interventions, and/or efficacious management of PV could aid in reducing PV-related HAs.

Concerning MDS, from 1990 to 2021, the worldwide incidence of MDS increased significantly by 99.3% because of population aging and growth.^[26]^ Besides population aging, the use of next-generation sequencing in diagnosing unclear cytopenias has also increased MDS cases.^[27]^ Cytopenias, including thrombocytopenia, neutropenia, and anemia, are the primary clinical complications of MDS.^[28]^ Cytopenias among MDS patients are associated with bleeding, serious infections, mortality, high utilization of healthcare services, emergency department visits, hospitalizations, transfusions, and/or other adverse consequences.^[28,29]^ Hence, the increase in MDS-related HAs in our study could partly be attributed to MDS complications. Prior research suggests that the outcomes of MDS patients may improve by managing MDS complications.^[28,30]^ Accordingly, this may also decrease the rate of HAs. Still, the links between MDS complications and rates of healthcare service utilization have not been investigated in the literature^[28]^; thus, further research is needed to explore these links.

The increase in the rate of MDS-related HAs could also be related to the multi-morbidity of elderly patients with MDS, the complexity of MDS management, and MDS heterogeneity, as the literature showed that these factors result in improper utilization of healthcare resources and raise the burden on healthcare systems.^[31,32]^ Moreover, a lack of an effective standard MDS treatment plan or therapeutic strategies may also contribute to an increase in the rate of MDS-related HAs; an earlier study revealed a significant increase in mortality rate, bleeding complications, and HAs for transfusions among certain symptomatic MDS patients due to a lack of a standard treatment plan for these patients.^[33]^ Another recent study also concluded that new therapeutic approaches are needed to improve the quality of life for MDS patients and optimize their management.^[34]^ Consequently, developing new therapeutic strategies and a standard treatment plan may aid in decreasing HAs for MDS.

This study showed that the age group 75 years and above accounted for 49.6% of the total number of HAs for PV and MDS. Besides, males accounted for 64.2% of the total number of HA episodes for PV and MDS, or 763,833 HA episodes, with a mean of 36,373 episodes per year. Additionally, over the duration of this study, the rate of HA for PV and MDS among males increased by 86.1% from 88.36 to 164.48 per 100,000 individuals; however, among females, it increased by 35.8% from 57.62 to 85.55 per 100,000 individuals. Our findings that elderly and male patients accounted for the bulk of HAs for MDS and PV could reflect the higher incidence of MDS and PV among elderly and male patients compared to other demographics, as reported by several prior studies.^[26,35,36,37–45]^ The higher HAs for MDS and PV among males may also be related to higher smoking^[46–48]^ and predominance of high-risk genes^[49,50]^ among males compared to females. Hence, it required the development of strategies to reduce HAs for MDS and PV, with a focus on older and male patients.

This study showed that the PV HA rate declined after 2014, especially in younger (15–59 years) and male patients. Potential explanations include the pervasive adoption of JAK2 testing and changes in diagnostic criteria, a shift toward outpatient management, coding changes, or misclassification. Masked PV, a variant of PV that is diagnostically challenging, received a growing amount of attention. Molecular and histopathologic features that are consistent with overt PV are exhibited by patients who do not meet the classic hemoglobin and hematocrit thresholds for PV as per earlier diagnostic guidelines but are classified as masked PV.^[51]^ Furthermore, an increasing emphasis has been placed on ambulatory and day-case care in the management of PV.^[43]^ Venesection is provided to decrease the hematocrit to <0·45 if it is not controlled at the time of acute thrombosis. In addition, cytoreductive therapy should be administered to control blood counts to the therapeutic target.^[43]^ Moreover, another expected cause for the observed decline in HA rate could be changes in ICD-10 coding practice or miscoding of PV episodes. However, this could not be confirmed without having clinical or laboratory data, which is not available in our dataset. Future research should examine PV-related hospitalization patterns using linked HES-outpatient and primary care data.

This study has limitations. The available HA data is based on finished consultant episodes, not unique admission episodes, where a new episode is reported when the same patient is transferred to a different provider or to another consultant. Therefore, a single HA could comprise multiple finished consultant episodes. This could have resulted in an overestimation of our HA rate estimates. Another limitation is that low-risk and high-risk MDS differ widely in biology, complications, and hospitalization patterns, and ICD-10 coding cannot distinguish risk between them. Therefore, HA rate estimates in this research represent aggregated HAs across MDS risk categories. Future research should utilize risk-stratified data (linkage with registries) to enhance the ability to examine HAs stratified by MDS risk category.

## 5. Conclusion

This study showed a significant increase in HAs for PV and MDS. The increase in HA among males was higher, and MDS specifically exceeds PV. This could be attributed to cytopenia complications, advances in diagnosis and treatment, the rise in incidences of MDS and PV, population growth, and aging. Besides, demographics influence the HAs for PV and MDS, with elderly and male patients accounting for the bulk of episodes. Thus, early diagnosis, interventions, and management are recommended to reduce HAs for PV and MDS, with a focus on older and male patients. Given that PV and MDS management has shifted toward outpatient management, this could indicate that HAs are driven by disease complications rather than inpatient disease management. This should be examined in future research utilizing linked HES-outpatient and registry data to examine this hypothesis.

## Acknowledgments

We would like to acknowledge Princess Nourah bint Abdulrahman University Researchers Supporting Project number (PNURSP2026R483), Princess Nourah bint Abdulrahman University, Riyadh, Saudi Arabia.

## Author contributions

**Conceptualization:** Richard Ofori-Asenso.

**Data curation:** Alaa A. Alsharif, Abdallah Y. Naser.

**Formal analysis:** Abdallah Y. Naser.

**Funding acquisition:** Alaa A. Alsharif.

**Investigation:** Alaa A. Alsharif, Abdallah Y. Naser, Richard Ofori-Asenso.

**Methodology:** Alaa A. Alsharif.

**Project administration:** Alaa A. Alsharif.

**Resources:** Alaa A. Alsharif, Abdallah Y. Naser.

**Software:** Abdallah Y. Naser.

**Supervision:** Alaa A. Alsharif, Abdallah Y. Naser.

**Validation:** Alaa A. Alsharif, Abdallah Y. Naser, Richard Ofori-Asenso.

**Visualization:** Alaa A. Alsharif, Abdallah Y. Naser, Richard Ofori-Asenso.

**Writing – original draft:** Alaa A. Alsharif, Abdallah Y. Naser, Richard Ofori-Asenso.

**Writing – review & editing:** Alaa A. Alsharif, Abdallah Y. Naser, Richard Ofori-Asenso.
